# The Cambodia Research Consortium: expediting research for malaria elimination with the emergency response to artemisinin resistance framework

**DOI:** 10.1186/s12936-015-1062-z

**Published:** 2016-01-04

**Authors:** Sara E. Canavati, Harriet L. S. Lawford, Bayo S. Fatunmbi, Dysoley Lek, Rithea Leang, Narann Top Samphor, Arjen M. Dondorp, Rekol Huy, Walter M. Kazadi

**Affiliations:** Department of Clinical Tropical Medicine, Faculty of Tropical Medicine, Mahidol University, Bangkok, Thailand; The National Center for Parasitology, Entomology and Malaria Control, Ministry of Health, Phnom Penh, Cambodia; Emergency Response to Artemisinin Resistance, WHO Representative Office in Cambodia, Phnom Penh, Cambodia; Mahidol Oxford Tropical Research Unit, Faculty of Tropical Medicine, Mahidol University, Bangkok, Thailand

**Keywords:** Malaria elimination, Operational research, Cambodia

## Abstract

This commentary offers insight into how to best address barriers that may hinder the translation of malaria research findings into policy. It also proposes viable methods of implementing these policies in Cambodia. Currently, a wide range of malaria research is being conducted by in-country stakeholders, including Cambodia’s National Programme for Parasitology, Entomology and Malaria Control’s (CNM), non-governmental organizations, and academic institutions. Coordinating research amongst these partners, as well as within the Ministry of Health, is a challenge. Results are rarely disseminated widely and seldom inform programme and policy decisions. CNM and its research partners have severely limited access to each other’s databases. This lack of accessibility, timeliness, engagement and cooperation between CNM and its partners greatly impacts overall research efficiency in this field, and is stifling innovation both within and beyond CNM. Cambodia has set a goal to eradicate all forms of malaria by 2030. As countries approach the elimination phase, there is a greater need for sharing research-generated evidence amongst partners, in order to ensure that appropriate and impactful activities are conducted. The Cambodian Research Consortium was established to serve as a framework for partners, stakeholders and researchers to share research projects, information and results, and to promote the goals of CNM. The sharing of malaria research results will help to inform prevention, control and elimination activities in the country. It will also determine and address the country’s operational research needs, and could potentially become a framework model to be used in other countries aiming to transition from malaria control to elimination.

## Background

A major challenge confronting low and middle income countries is optimizing high quality information to design policies [1]. The health component of the recently launched sustainable development goals (SDGs) by the United Nations—ensure healthy lives and promote wellbeing for all at all ages is predicated on the principles of Universal Health Coverage (UHC) [2]. To underscore the importance of health systems research and its linkage with health programming, a regional meeting on Applied Research on Health Policy and Systems to support UHC, held 26–27 November by the WHO Regional Office for the Western Pacific in Manila, Philippines, recommended as follows: “making the health systems and policy research agenda an integral part of multi-year national health plans; facilitating the development and strengthening of ‘process’ and platforms for institutionalizing the relationship between researchers and policy-makers; improving accessibility and quality of routinely collected data for health systems and policy research; some suggestive actions to improve ‘credibility’ and ‘relevance’ of health systems and policy research to improve its utilization; and institutional capacity building in health systems and policy research” [3].

In Cambodia, the number of malaria cases has fallen more than 81 % since 2009 [4] with this success attributed to significant progress in scaling up malaria control interventions, such as insecticide-treated bed nets (ITNs), and the introduction of the Village Malaria Worker (VMWs) programme to improve access to malaria diagnosis and treatment. However, limited structural and collaboration mechanisms have been invested in using research results to inform national malaria policies as well as the implementation of interventions in Cambodia. Currently, a wide range of malaria research is being conducted by in-country stakeholders, non-governmental organizations, and academic institutions. However, coordinating this research has been a challenge and results are often not widely disseminated and seldom inform programme and policy decisions. There are significant limitations in the accessibility, timeliness and documentation of the National Programme for Parasitology, Entomology and Malaria Control (CNM) partners’ data, which is reducing research efficiency and stifling innovation and translational research both within and beyond CNM.

These limitations represent a high opportunity cost in terms of the learning and innovation foregone, as well as a failure to fully render the public goods intended by the funders of the CNM system and the research donors. Therefore, a nationally-owned and managed research agenda to facilitate the use of this research, identify programme and research gaps and take into account national priorities to help accelerate Cambodia in moving towards elimination, is required. This will serve to both, strengthen in-country research activities and ensure research is orientated towards the country’s needs and inform opportunities for policy change.

In order to effectively support development and implementation of malaria elimination strategies, the CNM Taskforce for Research was established in January 2014, along with several taskforces under the CNM Sub-technical Working Groups (STWG) within the Ministry of Health (MoH)—Technical Working Group. The CNM Taskforce for Research aims to review and approve research proposals, oversee progress made on studies, collaborate with technical partners, disseminate research results and facilitate the translation of evidence into policy and practice where relevant. Due to several limitations, the CNM Taskforce for Research was not able to fulfill all of its aims, and thus the Cambodian Research Consortium was established. On 09 May 2014, the CNM STWG ratified the establishment of the Consortium, with technical and financial support from the Emergency Response to Artemisinin Resistance (ERAR), as a means to develop a research agenda for malaria elimination in Cambodia as well as to accelerate priority research activities. Action 8 in the ERAR Framework for Action 2013–2015 stipulates the need to fast track priority research and refine tools for containment and elimination [5]. The framework highlights the importance of sharing and disseminating information and lessons learned both nationally and regionally [5, 6].

The role of the Cambodia Research Consortium is described in detail along with the processes it incorporates aims to ensure that malaria research in Cambodia will be fully and easily accessible to CNM and its partners for policy development. The document also includes the partner landscape in current malaria research activities in Cambodia and lists research needs prioritized in 2014 to progress toward the goal of malaria elimination in Cambodia. The following sections of this commentary describe the inception and functioning of the Consortium within CNM and it ends with a summary of the discussion, conclusions and recommendations raised by STWG members at workshops held on 2–3rd June 2014 and 17th September 2014.

## Rationale for the Cambodia Research Consortium (CRC)

Practitioners and academics have investigated how evidence generated from research can be used to inform policy, and various strategies have been developed to ensure the timeliness and relevance of research in developing countries [7–9]. The Commission on Health Research for Development, an initiative to improve health and development in developing countries, recommended the establishment of the Essential National Health Research (ENHR), which encouraged countries to develop a sustainable research capacity to identify and prioritize their national research requirements to improve health, and link this with global efforts. Similarly, the web-based project ‘Getting Research into Policy and Practice’ (GRIPP) initiative, funded by the Department for International Development (DFID), aims to maximize the impact of research by documenting steps taken by researchers to complete case studies [10]. With relevance to artemisinin resistance, the Fogarty International Center and the National Institute of Allergy and Infectious Diseases of the U. S. National Institutes of Health held a conference in November 2010 with stakeholders, including scientists from countries threatened by malaria resistance [11].

With regards to malaria, a number of initiatives and consortiums have been created to enable the implementation of evidence-based strategies for achieving malaria control and elimination. On a global scale, the malaria Eradication Research Agenda (malERA) initiative has developed research and development priorities to identify knowledge gaps and tools in existing research agendas which complement existing research agendas aimed at malaria control [12]. The Malaria Eradication Scientific Alliance (MESA) has followed on from malERA and is working with multiple actors in malaria elimination to fulfil its three principle functions of (1) providing a platform for an evidence-based approach to global malaria eradication; (2) create knowledge management tools; and (3) accelerate research [13]. MESA-Track enables malaria research project to be shared worldwide. Regionally, the US Centres for Disease Control and Preventions (CDC) is leading a consortium of malaria partners aiming to eliminate indigenous cases of malaria on the island of Hispaniola (including Haiti and the Dominican Republic) by 2020 [14]. The Haiti Malaria Elimination Consortium (HaMEC) is being formed though this grant and will work to assist the countries of Hispaniola to develop, adopt, and implement an evidence-based strategy and operational plan for achieving malaria elimination [14]. Similarly, the Asia Pacific Leaders Malaria Alliance (APLMA), which is formed from the Asia Pacific Heads of Government, have agreed to the goal of an Asia Pacific free of malaria by 2030 and they have developed the Leaders’ Malaria Elimination Roadmap to establish a technically robust, strategically coherent and regionally coordinated approach to malaria elimination [15]. It is hoped that this roadmap will encourage a coordinated regional response, enabling the improvement in the quality and accessibility of key commodities and services in the region as well as sustained financing to see elimination through [15].

Research consortiums have also been developed for other diseases, such as HIV/AIDS and Tuberculosis (TB). For example, the TB Clinical Diagnostics Research Consortium (CDRC), inter-disciplinary consortium of scientists and clinicians with expertise in TB diagnostics, clinical trials, and international studies, was established in 2009; they collaborate closely with other TB research networks and consortia with the shared goal to identify better diagnostic tools and strategies for TB [16]. Lastly, the amfAR Research Consortium on HIV Eradication (ARCHE) supports collaborative teams of biomedical researchers exploring strategies for eradicating HIV infection [17].

However, despite these and other initiatives to ensure research findings are utilized, there remains a chronic gap from completing research and translating research findings into policy and practice. Questions have been raised as to why the gap between research and what it is done with it is so large [18, 19], with previous failures to translate research into policy and practice being attributed to a lack of ownership and not involving key policy and decision makes from the start [19]. The use of research has been found to increase if it is orientated towards the user’s needs [20, 21] emphasizing the need for continuous communication amongst national programmes and their partners.

Recently in Cambodia, bottlenecks to research have existed with some partners unable to conduct already funded projects due to rejections by the National Ethics and Human Rights Committee (NEHCR). When this was questioned, NEHCR responded by emphasizing that if proposals are not considered to be relevant to Cambodia, or if a project involves importing and testing something from abroad, it will be rejected. The CRC proposes to avoid such problems by collaborating closely with NEHCR. At a workshop in June 2014, NEHCR stated that if the CRC submit a letter of support along with a proposal, stating that it is a research priority and it is needed, this will be taken into consideration when they make their decision. Bringing key stakeholders involved in malaria research in Cambodia together under the Consortium provides a transparent platform for the processing of proposals, information sharing and encourages the use of research findings for translation into policy with a common goal and mutual benefits and trust.

## Objectives of the Cambodian Research Consortium

The CRC framework presents a detailed systematic approach to be adopted by all partners conducting malaria research in Cambodia. It establishes policies and procedures to be followed in order to ensure that malaria research is useful to the national programme and is impactful in practice for the malaria elimination goal.

CNM obtained consensus from multiple stakeholders to set up the objectives, structure and policies laid out under the CRC with an aim to improve coordination and own the evidence generated out of malaria research in country for policy making.

The objectives of the Cambodian Research Consortium are to:

- Coordinate all research related activities, including defining the research needs, review and approval of research proposals and mapping of all research studies;
- Support programme (CNM/Provincial Health Department/Operational District/Health Centre level) capacity building with support from research partners for the execution and scale-up of research projects;
- Communicate with NEHCR to facilitate the ethical approval process;
- Facilitate timely use of research findings to inform programme action including preparation of policy briefs, updating strategies, guidelines, and standard operating procedures, and promote information sharing among research stakeholders, including the establishment of a research database of CNM approved studies CNM, dissemination of findings, including drafting of scientific publications for peer-reviewed journals and peer-reviewed publications.

## Structure of the CRC

**Fig. 1:**
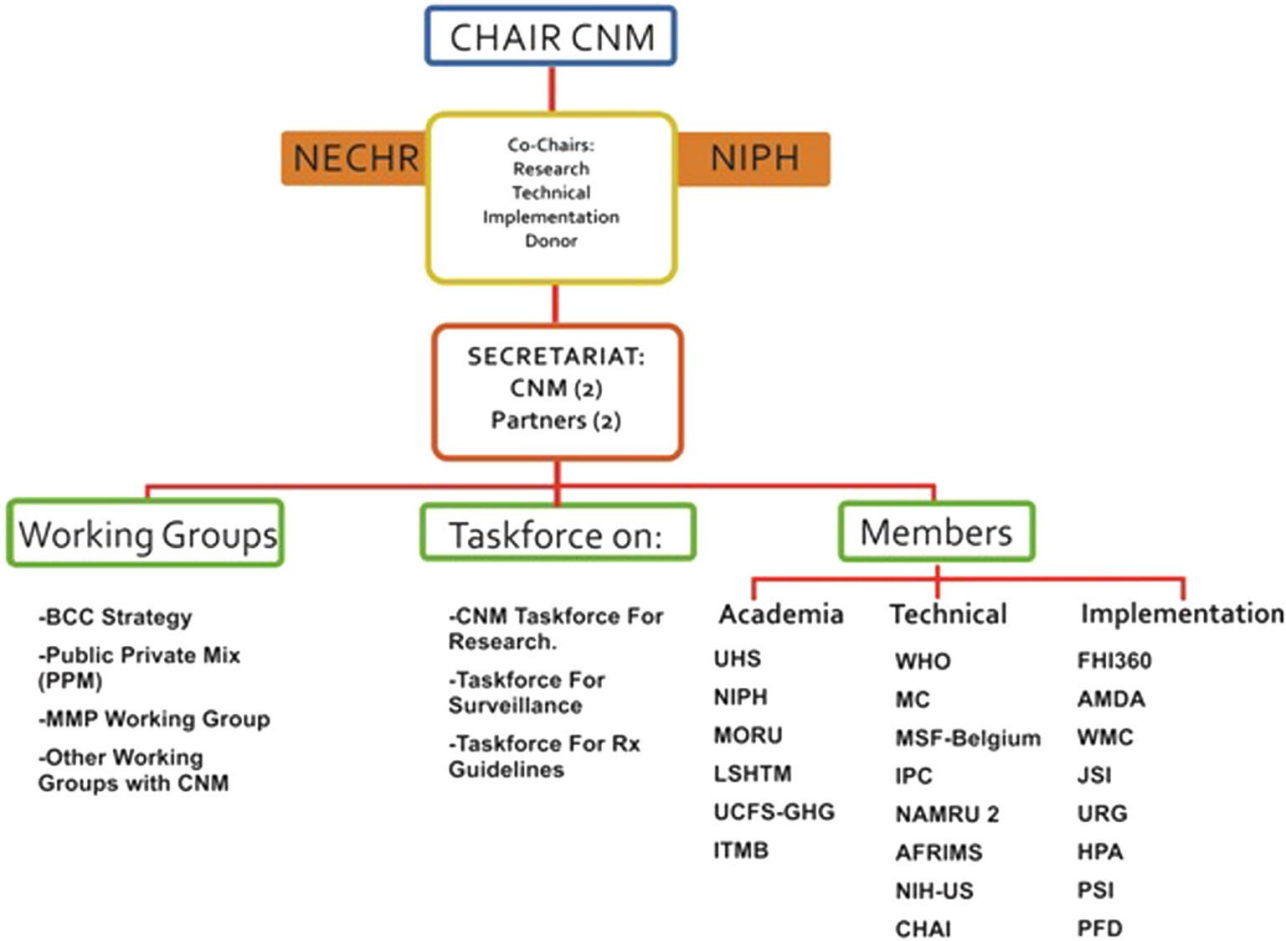
Structure of the Cambodian Research Consortium. The Cambodian Research Consortium is inclusive of CNM and its relevant departments, the Departments of Health, and the partners involved in malaria research and activities (academia, technical, implementation and donors)

**Fig. 2:**
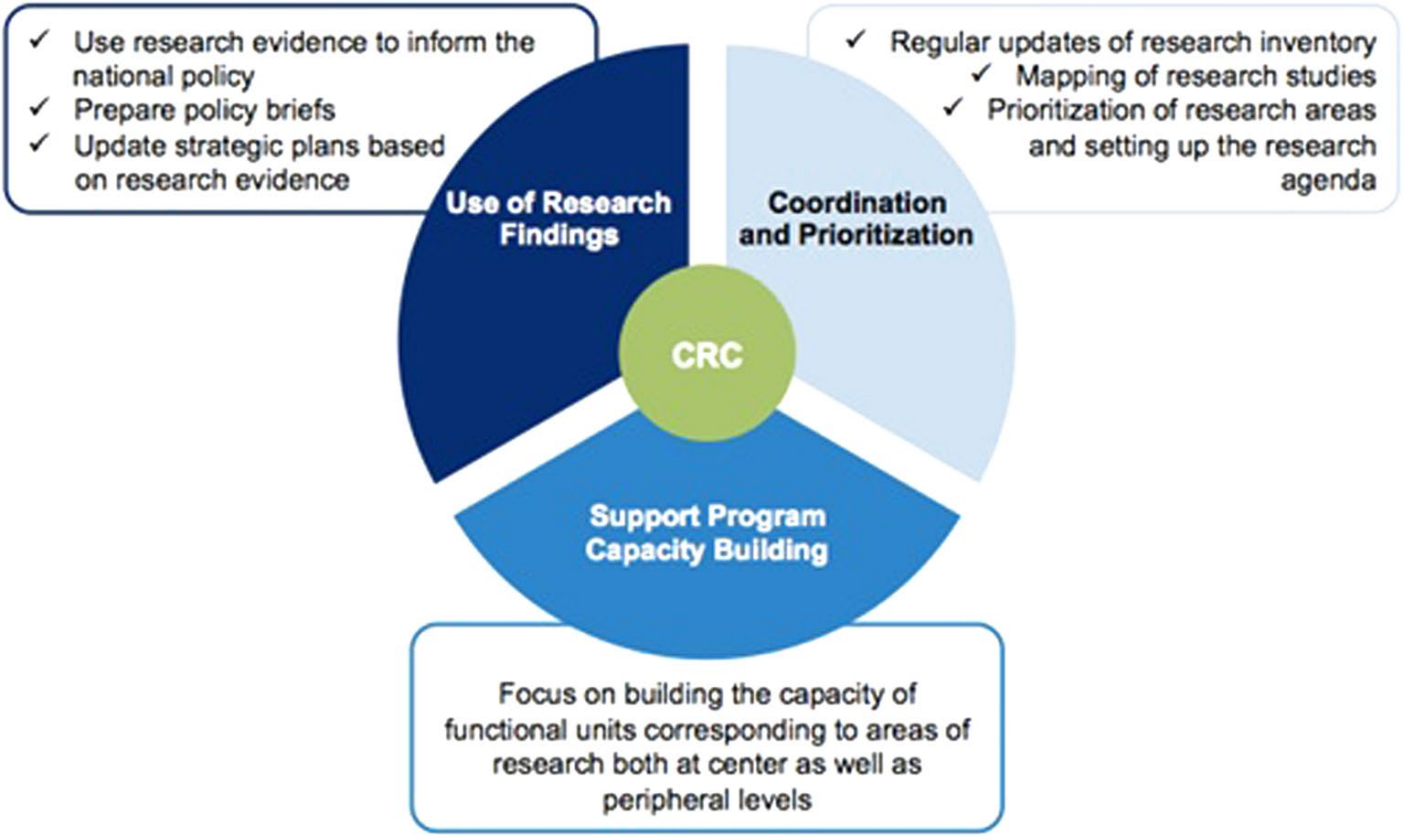
Strategic functions of the CRC. The strategic functions of the Cambodian Research Consortium have been categorized into three sub-groups, the roles of which are to coordinate within CNM and among partners, allow information sharing and enable the translation of research to inform policy

In order to obtain maximum input from and involvement of partners, the CRC is inclusive of CNM and its relevant departments, the Departments of Health, and the partners involved in malaria research and activities (academia, technical, implementation and donors) (Fig. 1). The strategic functions of the CRC have been categorized into three sub-groups (Fig. 2), the roles of which are to coordinate within CNM and among partners, allow information sharing and enable the translation of research to inform policy. This will not only encourage capacity building for CNM, but also ensure that projects are both specific to Cambodia and in-line with the national programme.

## Application process in the Cambodian Research Consortium

Submitting protocols through the CRC allows members to ensure that planned research aligns with the needs of CNM. It also expedites the process of ethical review as the Consortium can communicate with NEHCR and check all supporting documents are included. The application process can be described in three steps:

Step 1: Partner submits protocol, plans and other supporting documents (see next section);

Step 2: The CRC reviews the complete application to ensure that proposed research is aligned to the national strategic plan, prioritized research needs and conforms to the guidelines and standards set by the CRC;

Step 3: If proposed research meets criteria, then the Chair assigns a respective unit for research. If proposed research does not meet criteria; then Chair will send feedback to partner.

**Fig. 3:**
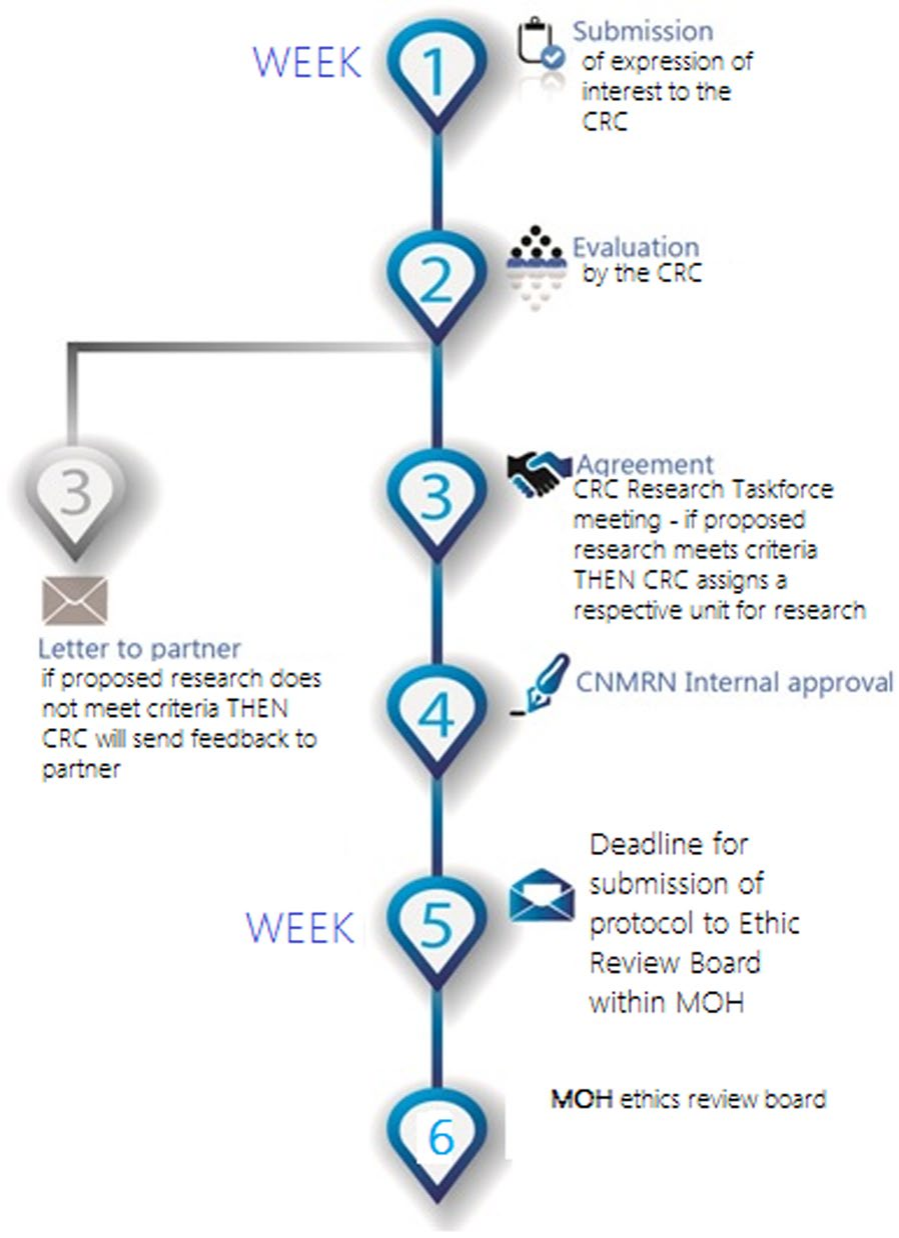
The schematic of the research application submission and review process of CRC. Timeline that describes a 6-week process from the submission of documents to the Cambodian Research Consortium until ethical review by the NEHCR

The related schematic outline can be seen in Fig. 3.

In order to make sure that this is a timely procedure, CRC members developed a timeline that described a six-week process from the submission of documents to the CRC until ethical review by the NEHCR. The CNM Research Taskforce will meet 2 weeks after an expression of interest has been submitted to CRC. Following that, 2 weeks is given for feedback and approval before submission to the ethics review board. Overall the process from the submission of expression of interest to the CRC to the MoH ethics review outcome is expected to take 6 weeks.

### Supporting documents required by the Cambodian Research Consortium

Partners are expected to make a formal application to the CRC to get support from CNM for their research studies. The application requires that the following documents are submitted:

- Screening checklist: this checklist serves as a summary document for protocols that are being submitted to the CRC. It details who will be involved in the study (PI and Co-Investigators), the study title, research type, study duration, participant characteristics and what supporting material is being submitted.
- Full protocol: including background, method, objectives and ethical considerations, as well as consent forms, dissemination plans and CVs of investigators.
- Data sharing agreement: this is completed between a partner and CNM before data owned by CNM is shared. The agreement states what data is required, who it will be shared with and terms and conditions for the use of the data.
- Capacity building plan: one of the key objectives of the CRC is to support capacity building of the national programme, its respective units and staff both at the central and provincial level. Partners are asked to include in their application how they will contribute to the capacity of human resources and infrastructure of research activities, how they will sustain and support the programme, whether they intend to scale up their research project, and if so, how.

### A platform for coordination of research

In light of national programmes now re-orientating their strategies from malaria control towards malaria pre-elimination and elimination, there is an urgent need to prioritize the operational research agenda in the Greater Mekong Sub-region (GMS). The CRC is a platform for partners, stakeholders and researchers to share research information and results to accelerate towards the goal of malaria elimination in Cambodia. Sharing research results will help to inform control and elimination activities in the country as well as determine and address the country’s research needs.

The main responsibility of the CRC is to coordinate all the malaria-related research activities in Cambodia, including the review of research proposals and the mapping of research studies conducted by partners to ensure coordination and relevance of research. By involving a large network of stakeholders, including policy makers and donors, the CRC can better understand and make use of different partners’ expertise. The CRC will maintain a database which compiles partners’ data from previous and ongoing malaria research. This will enable all partners and stakeholders to have access to evidence from research, including tested malaria interventions, and allow CNM to benefit from capacity building support. The CRC has allowed CNM to take a leadership role in the sector-wide research effort, highlighted by the WHO [9, 22, 23], to play a pivotal and catalytic role in developing the first ever research national consortium for malaria elimination.

### Data sharing

A review of minutes taken at two workshops held with the STWG members in June and September, 2014, identified questions and concerns related to the Consortium that had been raised by participants and needed to be addressed. These are important if similar consortiums are to be established in other countries. One topic that prompted much discussion was data sharing. It was highlighted that research institutes prefer to publish without sharing results beforehand and, therefore, sharing data was a sensitive issue for some partners, particularly regarding the confidentiality of information and results shared prior to publication. The CRC confirmed that preliminary information would be shared within CNM and the Consortium only to prevent any conflicts of interest.

Additionally, there were concerns regarding the timeliness of information sharing, particularly at the malaria pre/elimination stages where information is most needed. Means to ensure continued momentum for sharing and updating data in the research inventory should be established in order to facilitate the development of evidence based national guidelines. Such measures will include regular meetings between Consortium members to discuss ongoing and upcoming research, which will be entered by a designated individual at CNM.

### Ownership, alignment and harmonization

The Paris Declaration on Aid Effectiveness emphasizes on “Ownership, Alignment and Harmonization”. The CRC places heavy emphasis on capacity building as part of building country-ownership as well as alignment and harmonization of research. How to facilitate capacity-building was discussed and partners shared their experiences from within their own organizations. Capacity-building examples included training staff in other diseases, involving provincial level staff in dissemination activities and giving classes in technical writing in English to allow project staff to present their own data. Additional suggestions included the translation of preliminary results into field interventions and encouraging national representation at international meetings, not only enabling capacity building within CNM, but also commencing the process of using evidence to inform policy. As mentioned above, the capacity building plan is the fourth supporting document required by the CRC from CNM’s research partners. This plan ensures that the capacity building is a core component of all research projects. In addition, WHO/TDR has developed a capacity building strategy which was recommended as a good reference for future projects [24].

## Discussion

When discussed with partners, there was a consensus that the CRC will make working with CNM easier and more attractive, facilitate the ethical approval process and ensure that the structure of CNM is more transparent, whilst also keeping partners informed of CNM’s activities. Developing a national platform will allow partners to present the results of their research and enable partners to be in contact with each other through CNM. Active participation from CNM was encouraged, particularly with CNM staff conducting site visits and joining studies or helping to run them.

Partners also emphasized the need for the CRC to be a simple process that can be facilitated in CNM and can be applied to CNM and partners. CNM has the most number of partners regionally and 4 years of artemisinin resistance (AR) containment experience, yet a lot of information remains unpublished or published incorrectly. Technical reports and peer-review journal articles should be a deliverable from each project.

The feedback from the two workshops held with the CRC was very positive, with all partners supporting the establishment of the Consortium and the processes it will facilitate. Reviewing the minutes from the workshops provided useful information about what can be improved in the Consortium and what lessons can be transferred to other countries.

Since the CRC was launched in June 2014, the Consortium remains under the leadership of CNM’s director and the CNM Health Research Unit. Guidelines and supporting documents distributed to partners have been followed. The CNM has been able to gain more country-ownership of research conducted in the country. The bi-monthly meetings organized by CNM and funded by WHO have been taking place. In these meetings, researchers share progress of current studies as well as preliminary findings. These meetings have also been instrumental for creating a greater dialogue between CNM and partners on research priorities for Cambodia in the context of Multidrug Resistant malaria and malaria elimination. In addition, the Consortium has been very appealing to other countries in South East Asia, as they have shown interest in establishing a similar structure within their national programmes. The CRC has faced a number of challenges, such as internal and external coordination, commitment to the vision, availability of resources as well as other competing agendas. In order to address these challenges, the CRC has intensified one-to-one in-country dialogue with partners to enhance the coordination of research projects as well as to ensure the resources needed by CNM to facilitate and conduct research are in place. The research agenda of the CNM has been established and re-enforced in the recently released Cambodia Malaria Elimination Action Framework.

## Conclusion

The ERAR Framework through the CRC will serve as a framework for partners, stakeholders and researchers to share research projects, information and results to promote the goals of the CNM. Sharing these results will help to inform malaria control, prevention and elimination activities in the country as well as determine and address the country’s operational research needs. ERAR hopes to encourage the translation of evidence generated form malaria elimination research to inform policies as Cambodia moves from malaria control to elimination, and thereby enable Cambodia to be a model country to share its experiences with others in the region.
